# Diet Acceptance and Utilization Responses to Increasing Doses of Thymol in Beef Steers Consuming Forage

**DOI:** 10.3390/ani15243637

**Published:** 2025-12-17

**Authors:** Emma P. Fukuda, Jordan P. Suter, Russell W. Jessup, Merritt L. Drewery

**Affiliations:** 1 Department of Agricultural Sciences, Texas State University, 601 University Dr., San Marcos, TX 78666, USA; 2 Department of Crop and Soil Sciences, Texas A&M University, 400 Bizzell St., College Station, TX 77843, USA; rjessup@tamu.edu

**Keywords:** beef cattle, essential oils, feed additives, methane, secondary plant metabolites, sustainability

## Abstract

Thymol is an antimicrobial terpene with potential as a feed additive for cattle; however, in vivo data describing the response of forage-fed beef cattle to the increasing provision of thymol is limited. As thymol may affect palatability and exert antimicrobial effects on rumen microbes, defining its effects in vivo is critical to inform adoption. Accordingly, the objectives of this study were to evaluate cattle acceptance of thymol and characterize the effects of increasing thymol doses on diet utilization and ruminal fermentation, with the overarching goal of identifying a maximum tolerable dose for beef cattle. Two 4 × 4 Latin Square experiments were conducted using beef steers consuming forage and providing thymol dosed on alfalfa. Experiment 1 assessed acceptance of thymol at increasing concentrations (0, 110, 220, and 330 mg/kg intake), and experiment 2 assessed diet utilization and fermentation in response to one of four thymol doses: 0, 120, 240, and 480 mg/kg intake. For experiment 1, thymol dose did not affect treatment or forage intake (*p* ≥ 0.17). For experiment 2, thymol did not linearly or quadratically affect (*p ≥* 0.28) forage organic matter (OM) intake or OM digestibility. There were also no linear or quadratic effects (*p ≥* 0.09) on ruminal ammonia-N, volatile fatty acids, or pH. These data indicate that thymol can be provided to beef cattle at up to 330 mg/kg intake and 480 mg/kg intake without negatively impacting acceptance and diet utilization, respectively.

## Simple Summary

Plants naturally produce protective compounds, called secondary plant metabolites, in response to stress. These compounds, including essential oils and terpenes, have antimicrobial, antioxidant, and anti-inflammatory properties. There is a need to research how secondary plant metabolites might serve as natural feed additives in livestock production. Thymol is a terpene found in oregano and thyme oil that can inhibit harmful microbes; however, if livestock are fed too much, it could also disrupt the beneficial microbes that cattle require for digestion. In this study, we tested how beef cattle responded to different amounts of thymol being included in their normal diet. We evaluated whether cattle would readily eat thymol and whether thymol affected digestion and fermentation in the stomach. Our results indicate that cattle willingly ate feed containing thymol and that digestion and ruminal fermentation were not statistically affected by thymol, even at the highest amounts tested (330 and 480 mg/kg, respectively). However, we observed numerical trends in acceptance and fermentation measures that should be considered before adoption. These data suggest that thymol can be fed to cattle in the amounts tested, demonstrating its potential as a feed additive and helping establish a maximum tolerable level for beef cattle consuming forage.

## 1. Introduction

Secondary plant metabolites (SPMs) are produced by plants in response to environmental stressors [1,2], thereby giving them antimicrobial [3], anti-inflammatory [4], antioxidant [5], and antiviral effects [6]. In recent years, humans have increasingly consumed or utilized SPMs, such as essential oils (EOs), to benefit from their natural restorative properties [7]. Many SPMs are present in the natural diet of various ungulates [8], and research on the benefits of dietary SPMs in cattle has illuminated their potential use as natural anthelmintic agents [9], antibiotics [10], for improving the meat quality and fatty acid profile of beef [11], as methane-mitigating feed additives [12,13,14,15], and as rumen modifiers to increase feed efficiency [16]. Due to increased antimicrobial resistance in ruminants [17] and consumer concern about antibiotic residues in meat, the demand for meat produced without traditional antibiotics is increasing in the Western world [10,18,19] and may be addressed by incorporating SPMs in livestock diets.

The SPM category is broad, containing many structurally diverse classes, including tannins, saponins, EO, terpenes, and flavonoids. Further, each of these classes contains numerous unique compounds with differing effects; therefore, it is important to distinguish between individual SPMs when comparing outcomes across studies. Terpenes represent the most diverse class of SPMs [1,20,21] and are the primary bioactive components of EO. Each EO has a terpene profile, which is often dominated by one to three terpenes, and low concentrations of others that each have a unique structure and function. Terpenes are responsible for the biological effects observed when EOs are consumed [3]. However, terpenes are not standardized within EOs and vary depending on many factors (e.g., climate, soil nutrient availability, and plant components) [1,2]. Accordingly, the previous literature reports contradictory biological effects of feeding EOs to livestock [3], which may pose a challenge to the adoption of EOs as feed additives. Therefore, it may be more effective and consistent to feed the bioactive components of EOs (i.e., terpenes) directly to cattle.

Terpenes exert antimicrobial effects primarily through disrupting microbial cell membranes [22]. Because rumen function depends on an active and diverse microbial community, high doses of dietary terpenes may suppress fermentation and impair nutrient utilization, underlining the importance of investigating dose-dependent effects. More specifically, low or moderate doses may support desired biological outcomes, while higher doses (≥500 mg/L batch culture of rumen fluid) could inhibit ruminal microbial activity [23,24], ultimately impairing animal performance. A clear understanding of this dose–response relationship is necessary to inform the use of terpenes as feed additives for cattle.

Despite extensive in vitro research on terpenes, a key knowledge gap remains in whether the doses that alter fermentation and microbial populations in vitro translate to tolerable and/or performance-inhibiting levels in vivo in beef cattle consuming forage. Existing in vivo research has primarily utilized modest inclusion rates, and it is unclear whether these approach the threshold beyond which performance may be sacrificed [25,26].

Thymol is a terpene that has antimicrobial activity similar to that of carvacrol, a constituent of oregano and thyme oil [26], but is available at a lower price point. Thymol has a well-characterized antimicrobial mode of action; it has a low molecular weight and is lipophilic, easily penetrating the phospholipid bilayer of microorganisms to inhibit growth of both Gram-negative and Gram-positive bacteria and archaea [26]. Although carvacrol and thymol share structural similarities, differences in their lipophilicity, stability, and interactions with microbes may influence their metabolism and functional effects in the rumen [27,28]. Previous research indicates thymol is a promising agent in ruminants for enteric methane mitigation [29], reduction in parasites and pathogenic bacteria [9,30], and rumen modification for increased efficiency [31], but these outcomes vary based on the dose provided and the basal diet or substrate used.

Studies assessing thymol in vivo in cattle are limited and have primarily evaluated modest levels that do not align with concentrations demonstrated in vitro to alter rumen microbial communities and/or fermentation products [32]. Further, because SPMs have strong aromatic or bitter sensory characteristics, cattle may not voluntarily accept higher doses [33], which would not be reflected in in vitro work.

Therefore, the objectives of this study were to evaluate cattle acceptance of thymol as a feed additive and to determine the effects of increasing doses of thymol on forage utilization and ruminal fermentation in cattle consuming forage. These objectives ultimately support the aim to establish a maximum tolerable dose of thymol for forage-fed beef cattle.

## 2. Materials and Methods

Experimental procedures involving animals were approved by the Institutional Animal Care and Use Committee at Texas State University (#8693). Animals were ruminally cannulated and healed from surgery for at least three months before initiation of procedures. Further, a different group of animals were used for experiment 1 versus experiment 2. Thymol was obtained in crystal form at 99% purity and stabilized using liquid nanocellulose to prevent volatilization upon feeding, and then was diluted with water to the target dose rate.

### 2.1. Experiment 1

Angus steers (539 ± 54 kg BW; ~25 months of age) housed individually in a partially enclosed barn and fitted with rumen cannulas (*n* = 4) were used in a 4 × 4 Latin Square design experiment to determine cattle acceptance of increasing doses of thymol as a feed additive. Procedures were adapted from previous research [34]. Steers were adapted to housing for 10 d before initiation of the experiment. Periods included a 3-d experimental phase where treatments were offered and 3-d washout where steers were fed a 1 kg cottonseed meal (CSM), a conventional protein supplement.

A basal diet of kleingrass (Table 1; 7.2% crude protein [CP], 76% neutral detergent fiber [NDF]) was provided at 130% of the previous 3-d intake daily at 0730 h. Treatments were 0, 110, 220, and 330 mg thymol/kg forage intake; this dose was based on the previous 3-d forage intake for individual animals and thymol was soaked into alfalfa cubes, which were offered daily (0730 h) at 0.20% BW in a separate feed pan within each pen. Treatments were offered during each experimental phase for 30 min, after which refusals were weighed.

### 2.2. Experiment 2

Angus steers (315 ± 25 kg; ~13 months of age) fitted with rumen cannulas (*n* = 4) were used in a 4 × 4 Latin Square experiment. Steers were housed in a partially enclosed barn in individual stalls and provided ad libitum water and a coastal blend of kleingrass and medio bluestem (3.4% CP, 69.9% NDF) at 130% of the previous 5 d average intake in addition to alfalfa cubes at 20% of hay intake from the previous day (Table 1) at 0730 h daily; alfalfa was provided to ensure N of the hay was not limiting to rumen microbes and as a carrier for thymol.

Steers received one of four treatments each period: a negative control with no thymol (CON), and thymol at either 120 (120-T), 240 (240-T), or 480 (480-T) mg/kg of forage (i.e., hay and alfalfa) intake from the previous day. This dose rate was comparable to the levels of thymol provided in the previous literature [35] in the form of thyme EO to concentrate-fed steers. Thymol was soaked into alfalfa cubes and offered in separate pans daily at 0730 h. Alfalfa/thymol refusals remaining after 30 min were directly infused into the rumen to ensure complete consumption.

Before initiation of the experiment, steers were adapted to their housing for 10 d. Each of the four periods was 28-d, with 8-d for treatment adaptation, 4-d for measurement of intake and digestion, 1-d for determination of ruminal fermentation, and 1-d for sampling subcutaneous fat to determine accumulation of terpenes in adipose tissue. Finally, 14-d were allocated at the end of each period for the carryover effect to allow terpenes in the adipose tissue to return to baseline. During the 14-d carryover, steers were fed ad libitum hay and 0.75 kg/d of soybean meal but did not receive alfalfa cubes or thymol. Hay refusals (orts) were collected and weighed daily.

Feed samples were collected on days 9–12 of each period, and orts were sampled on days 10–13. Fecal grab samples were collected on days 10–13 every 8 h, starting at h 0, with sampling time advancing by 2 h every day to represent every other hour of an entire day. Fecal samples were stored at −20 °C prior to thawing for further analysis.

Rumen fluid samples were collected on day 14 of each period at 0, 4, 8, 12, 16, and 20 h after feeding using a suction strainer from various locations within the rumen to obtain a representative sample. Directly after sampling, rumen pH was measured using a portable pH meter with a combined electrode (Fisher Scientific, Waltham, MA, USA). Rumen fluid was diluted as 8 mL of rumen fluid combined with 2 mL of 25% *m*-phosphoric acid and 9 mL of rumen fluid combined with 1 mL of 1 N hydrochloric acid and stored at −20 °C for subsequent analysis of volatile fatty acid (VFA) and ruminal ammonia-N concentrations, respectively.

Biopsy samples of subcutaneous fat (~1 g/sample) were collected from alternating sides of the tail head on day 15 of each period at 24 h after feeding using methods that have been previously outlined [36]. The sampling area was clipped, disinfected, and anesthetized before cutting a V-shaped incision using a surgical scalpel below the skin surface. Adipose tissue samples were snap-frozen in liquid N before storage at −20 °C.

### 2.3. Laboratory Analysis

Diet samples were collected in experiment 1, and diet, refusal (orts), and fecal samples were collected in experiment 2. Samples were maintained and analyzed separately for each experiment. Diet, refusal, and fecal samples were dried at 55 °C in a forced-air oven for 96 h and were allowed to air equilibrate before weighing for the determination of partial dry matter (DM). Diet and fecal samples were ground with a Wiley mill (Thomas Scientific, Swedesboro, NJ, USA) to pass a 1 mm screen. Diet samples were composited on an equal weight basis across days within a period. For experiment 2, refusal and fecal samples were also composited by steer across days within period.

Diet and fecal samples were analyzed for DM, organic matter (OM), N, neutral detergent fiber (NDF), and acid detergent fiber (ADF). For experiment 2, diet and fecal samples were also analyzed for acid detergent insoluble ash (ADIA), which served as an internal marker. Diet and fecal samples were dried at 105 °C for 24 h and allowed to air equilibrate in a desiccator before weighing for the determination of DM content. For analysis of OM, samples were combusted at 450 °C for 8 h and weighed to determine loss in dry weight. Nitrogen was analyzed using Dumas combustion, and CP was calculated as N × 6.25. Fiber characteristics (i.e., NDF, ADF) were measured non-sequentially using an Ankom Fiber Analyzer (Ankom Technology Corp., Macedon, NY, USA) with sodium sulfite and amylase omitted and without correction for residual ash. For experiment 2, residues of ADF were then combusted for 8 h at 450 °C for determination of ADIA.

Rumen fluid was collected from four locations within the dorsal and ventral sacs of the rumen using a suction strainer [37] for experiment 2; rumen fluid was thawed and centrifuged at 20,000× *g* for 20 min. VFA concentrations of ruminal fluid samples were measured according to [38]. Specifically, procedures used an Agilent 7890A gas chromatograph (Agilent Technologies, Santa Clara, CA, USA) equipped with a 30 m × 0.32 mm × 0.25 µm (i.d.) column (Alltech, Deerfield, IL, USA) with an initial column temperature of 80 °C held for 1 min, ramped to 185 °C at 15 °C/min, and then held for 2 min. Injector and detector temperatures were 250 °C. The column flow rate was 2 mL/min, and H_2_ was the carrier gas. Ruminal ammonia-N was measured using a UV-vis spectrometer with colorimetric procedures outlined in [39].

For determination of aroma volatile uptake in adipose tissue of animals consuming thymol (experiment 2), biopsy samples were weighed into glass vials with a Teflon septum lid along with 10 µL of 1,3-dichlorobenzene solution (2.5 µg/µL), which served as an internal standard. Samples were heated to 65 °C for 30 min on an electric heating block (VWR, Radnor, PA, USA). A solid-phase micro-extraction (SPME) portable field sampler (Supelco 504,831, 75 μm carboxen/polydimethylsiloxane, Sigma-Aldrich, St. Louis, MO, USA) was used for the collection of volatile compounds present in the headspace of the vials. Volatiles were eluted from the SPME after collection and separated with a gas chromatograph (GC; Agilent Technologies 7920 series GC, Santa Clara, CA, USA), where the sample was desorbed at 280 °C for 3 min. Samples were loaded onto a GC fitted with a column (30 m × 0.25 mm ID/BPX5 [5% phenyl polysilphenylene-siloxane] 1.0 μm, SGE Analytical Sciences, Austin, TX, USA) using He as the carrier gas at 1.0 mL/min. The GC temperature was 40 °C for 1 min and increased at a rate of 20 °C/min until it reached 250 °C. Compounds were identified and quantified with a mass spectrometer (Technologies 5975 series MS, Santa Clara, CA, USA) for relative identification using the NIST Chemical Library [40]. During this analysis, one adipose tissue sample was lost due to reasons unrelated to experimental treatment. As a result, there was not enough statistical power in the model to analyze adipose tissue volatiles associated with thymol consumption, which limited our ability to meaningfully interpret these data. Therefore, treatment means and measures of central tendencies for adipose tissue volatiles are presented in Supplemental Table S1, and these data are not discussed in the remainder of the manuscript.

### 2.4. Calculations and Statistical Analysis

For experiment 1, disappearance of treatments (i.e., amount of thymol-soaked alfalfa cubes consumed) was analyzed using the MIXED procedure in SAS v9.4. Terms in the model included treatment, period, day, and treatment × day with steer and treatment × period × steer as random terms. The repeated term was day with treatment × steer as the subject. The specified covariance structure was selected based on the Bayesian Information Criterion. The LSMEANS option was used to calculate treatment means.

For experiment 2, digestibility and fecal production were calculated as outlined in previous research [41], and ADIA was utilized as an internal marker to estimate fecal production. More specifically, fecal production was calculated as follows:

$$Fecal\;production,\;kg=\frac{DMI\times [{ADIA}_{d}]}{\left[{ADIA}_{f}\right]}$$

where DMI = dry matter intake, [ADIA_d_] = dietary ADIA concentration (% DM), and [ADIA_f_] = fecal ADIA concentration (%DM).

Statistical analysis of intake, digestion, rumen fermentation parameters, and adipose tissue aroma volatiles was conducted using the MIXED procedure in SAS 9.4 (SAS Inst. Inc., Cary, NC, USA). For intake and digestion, fixed terms were treatment and period with steer as the random effect. For fermentation parameters, fixed terms included treatment, period, hour, steer, and treatment × hour, with steer and treatment × period × steer included as random terms. Treatment means for intake, digestion, and fermentation parameters were calculated using the LSMEANS option and tested using linear and quadratic orthogonal contrasts.

## 3. Results

### 3.1. Experiment 1: Acceptance of Thymol

There was no effect of treatment × day (*p* = 0.51), treatment (*p* = 0.17), day (*p* = 0.40), or period (*p* = 0.26) on treatment (alfalfa cubes soaked in thymol) intake, which averaged 1.10 kg/d (Table 2). There was also no effect of treatment × day (*p* = 0.71), treatment (*p* = 0.18), or period (*p* = 0.35) on hay intake, which averaged 9.78 kg/d across treatments. However, there was an effect of day (*p* ≤ 0.01) on hay intake, in that steers consumed significantly more (*p* ≤ 0.01) hay on day 2 (10.4 kg/d) versus day 3 (9.15 kg/d), and the differences between day 1 and 2 (*p* = 0.09) and day 1 and 3 were statistical trends (*p* = 0.10).

### 3.2. Experiment 2: Forage Utilization and Rumen Fermentation

We did not observe linear or quadratic effects of increasing thymol dose on forage organic matter intake (FOMI), where FOMI for CON was 4.48 g/kg BW, and for 120-T, 240-T, and 480-T, it was 4.58, 4.63, and 4.74 g/kg BW, respectively (*p* ≥ 0.55; Table 3). We also did not observe a linear or quadratic response of supplemental organic matter intake (SOMI; *p* ≥ 0.23) or total organic matter intake (TOMI; *p* ≥ 0.47) across treatments. Total digestible organic matter intake (TDOMI) was also not linearly (*p* = 0.54) or quadratically (*p* = 0.31) affected by thymol dose; steers receiving CON consumed 3.15 kg TDOMI/d, 120-T was 3.01 kg TDOMI/d, 240-T was 3.24 kg TDOMI/d, and 480-T was 3.32 kg TDOMI/d.

There were no linear or quadratic effects of thymol dose on dry matter digestibility (DMD; *p* ≥ 0.22), where the average DMD for steers was 52.0% across treatments. We also did not observe linear or quadratic treatment effects on organic matter digestibility (OMD; *p* = 0.28) or neutral detergent digestibility (NDFD; *p =* 0.19), which averaged 56.7% and 63.0% across treatment groups, respectively.

**Figure 1:**
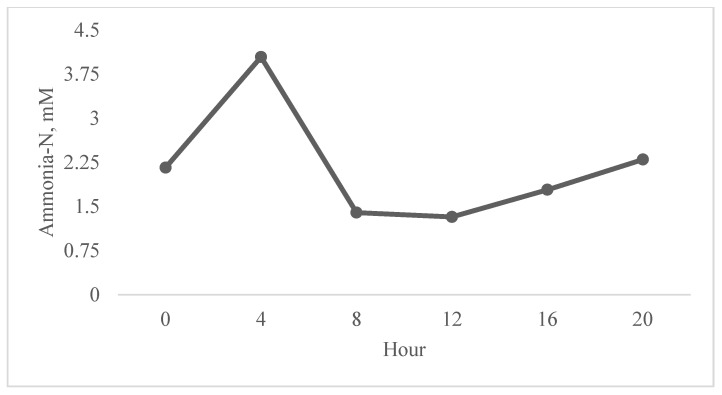
Average ruminal NH_3_-N concentrations of steers fed four graded levels of thymol (0, 120, 240, and 480 mg/kg intake) with hay and alfalfa cubes. No effect of treatment × hour (*p* = 1.00). No linear effect of treatment (*p* = 0.83). Quadratic trend of treatment (*p* = 0.09) with lowest NH_3_-N concentration at 120 mg thymol/kg intake. Effect of time (*p* ≤ 0.01). SEM ± 0.21.

There was no treatment × hour interaction effect on ruminal ammonia-N concentrations (*p* = 1.00; Table 4). There was also no linear effect of treatment on ruminal ammonia-N concentrations (*p* = 0.83). Ruminal ammonia-N was significantly affected by hour, with the highest concentration observed at h 4 (4.40 mM, *p* ≤ 0.01; Figure 1); there were no linear or quadratic effects of hour on ruminal ammonia-N (*p* = 0.10).

**Figure 2:**
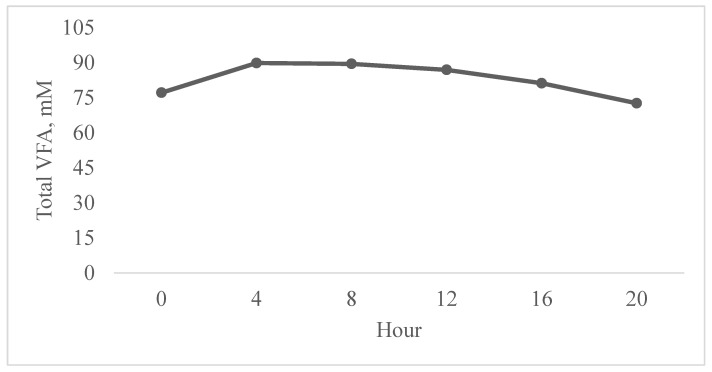
Average volatile fatty acid (VFA) production of steers fed four graded levels of thymol (0, 120, 240, and 480 mg/kg intake) with hay and alfalfa cubes. No treatment × hour effect (*p* = 0.93) or linear or quadratic effect of treatment (*p* = 0.83). Effect of time (*p* ≤ 0.01). SEM ± 2.84.

There was no effect of treatment × hour on total VFA (*p* = 0.93), and there were no linear or quadratic effects of treatment on total VFA (*p* = 0.83). However, there was an hour effect for total VFA (*p* ≤ 0.01; Figure 2), with the highest VFA concentration observed at h 4 (89.84 mM). There were no linear or quadratic effects of hour on total VFA (*p* = 0.82).

There was no treatment × hour effect on molar proportions of acetate (*p* = 0.92), propionate (*p* = 0.86), or butyrate (*p* = 0.97). Molar proportions of acetate linearly increased as thymol dose increased, where CON steers had 74.8% acetate, 120-T steers had 74.9%, 240-T steers had 75.4%, and 480-T steers had 75.0% (*p* = 0.04). There were no linear or quadratic responses to treatment for molar proportions of butyrate (*p* ≥ 0.46), isobutyrate (*p* ≥ 0.79), valerate (*p* ≥ 0.12), or isovalerate (*p* ≥ 0.32).

There was a quadratic (*p* = 0.05), but not a linear (*p* = 0.21), effect of treatment on the acetate-to-propionate ratio (A:P), with the highest A:P for steers receiving 240-T (5.38; *p* = 0.05), where steers receiving CON, 120-T, and 480-T were 5.18, 5.20, and 5.21, respectively.

There was no treatment × hour effect on ruminal pH (*p* = 0.87), nor were there linear or quadratic effects (*p* ≥ 0.62). Average rumen pH across the thymol dosage was 6.61. There was an effect of hour on pH (*p* ≤ 0.01), where the lowest value was observed at h 8 (6.89), but there were no linear or quadratic effects of hour (*p* = 0.62).

## 4. Discussion

We conducted two experiments to evaluate cattle acceptance of thymol as a feed additive and to determine the effects of an increasing dose of thymol on diet utilization and ruminal fermentation in cattle consuming forage. Much of the existing literature related to thymol and broader EO provision to cattle has been conducted in vitro, and the doses utilized are likely too high to be practical in production due to the cost and potential palatability issues associated with aromatic compounds [42]. Additionally, in vitro responses may not be predictive of in vivo outcomes due to dietary- and microbial-dependent interactions [25,26]. Therefore, we provided practical doses of thymol to cattle consuming a basal forage diet.

In the acceptance trial (experiment 1), we dosed alfalfa cubes with graded amounts of thymol from 0 to 330 mg/kg intake and did not observe significant differences in treatment intake. It is worth noting, however, that we observed a numerical decline in acceptance of thymol-soaked alfalfa cubes at higher doses (330 mg/kg intake), suggesting that sensory characteristics of thymol may influence voluntary intake at higher inclusion levels. This is consistent with the recognized aversive organoleptic qualities of certain SPMs [33] and indicates that cattle may approach an acceptance threshold for thymol at 330 mg/kg intake.

There was also a day effect observed for the basal forage diet (i.e., greater hay intake on day 2 versus 3), which may suggest that factors unrelated to thymol, such as daily variation in environmental conditions or normal fluctuations in animal appetite and satiety, could contribute to variations in treatment disappearance, although we did not also observe an effect of day on treatment intake. Because thymol was dosed based on the previous 3-day forage intake, lower hay intake on a single day is not anticipated to have artificially inflated thymol provision and intake.

As alfalfa cubes, the carrier for thymol, were provided in a fixed amount (0.20% BW), alfalfa intake represented approximately 10% of total intake for the day. Therefore, if thymol were to be incorporated into a total mixed ration, it is unlikely that thymol provided at the same concentrations as researched here would numerically reduce intake due to the diluting effect of additional feed. However, thymol provided as an additive to a basal ration, as modeled here, may be constrained by a threshold approaching or slightly greater than the 330 mg/kg intake level we implemented.

In experiment 2, we provided a wider range of graded thymol doses to cattle consuming forage, from 0 to 480 mg/kg intake, on alfalfa cubes provided at 20% of intake, which represented a greater percentage of the total diet than in experiment 1. While we did not observe issues with treatment intake, we did not quantitatively measure it. More research, as described in experiment 1, should be conducted to determine the optimal thymol to alfalfa (or other feed carrier) ratio that cattle will tolerate. These efforts can inform product development for a thymol-containing feed additive to be delivered in pastured and extensively managed cattle. Further, conducting acceptance research with different basal forage types or seasonal conditions may clarify how interactions between diet and other environmental factors impact cattle acceptance of thymol.

In vitro research suggests thymol could inhibit rumen fermentation if provided at doses exceeding 500 mg/L rumen fluid [24,41,43,44], which may impair animal performance. Data from experiment 2 indicates that intake, digestion, and ruminal fermentation in beef steers consuming forage were not negatively impacted by thymol provided at 120–480 mg/kg intake. Assuming an average rumen volume of 80 L [45], our doses translated to 11 mg thymol/L rumen fluid (120-T), 22 mg thymol/L (240-T), and 44 mg thymol/L (480-T), which are lower than the documented inhibitory in vitro levels. In previous in vivo research, thyme EO was fed to Holstein steers consuming a total mixed ration without affecting rumen fermentation, intake, and digestion [35]; as thyme EO had a thymol concentration of 45%, the dose used in that study provided 225 mg thymol/kg DM, which is similar to the 240 mg/kg (240-T) treatment utilized in our research. Collectively, these data suggest that the antimicrobial potency demonstrated at high in vitro levels does not translate to inhibition of in vivo outcomes when thymol is provided at practical dose rates.

Condensed tannins, another SPM commonly researched to reduce methane production in cattle, often negatively affects digestion; this is likely due to the lowered abundance of fibrolytic microorganisms in the rumen [46,47]. Previous research indicates that certain fibrolytic microbes, which dominate the rumen of forage-fed cattle, are less sensitive to thymol and EOs than bacteria involved in starch and protein degradation [23,48]. Therefore, thymol may be a more viable SPM for methane suppression and/or to modulate the rumen environment than condensed tannins, as our findings indicate it does not inhibit forage utilization.

Ruminal ammonia-N and total VFA concentrations were not affected by thymol at the doses provided in this study. This agrees with other research [32,35], where there was also no effect of either thyme EO or thymol on these fermentation parameters. Ruminal ammonia-N is associated with microbial growth and efficiency and N utilization [49], and VFA are the primary energy derivatives for ruminants [50]; therefore, these fermentation parameters directly translate to animal performance. Rumen pH was also not affected by thymol, which agrees with other findings [35]. Therefore, despite having strong documented antimicrobial effects [24], thymol provided at up to 480 mg/kg intake in our study did not negatively impact ruminal fermentation. Taken together, these findings suggest that thymol provided up to 480 mg/kg intake does not disrupt the primary fermentation pathways governing nutrient utilization in cattle consuming forage.

In vitro research has demonstrated that the effect of thymol on molar proportions of VFA is driven by pH, where a neutral rumen pH (7) corresponds to a higher A:P than a rumen with a slightly more acidic pH (5.5) in the presence of thymol [51]. There was an effect of thymol on the rumen pH of rams, where rams consuming a low-concentrate diet and fed 300 mg thymol/kg DMI had higher pH than rams consuming a low-concentrate diet and receiving no thymol or rams consuming a high-concentrate diet and receiving 600 mg thymol/kg DMI [31]. In the same study, there was decreased ruminal ammonia-N for rams fed a low-concentrate diet with 600 mg thymol/kg DMI compared to rams consuming either a low- or high-concentrate diet and receiving 300 mg thymol/kg DMI [31]. This suggests that both the basal diet and thymol dose affect rumen fermentation, which contrasts with our data. However, while rams in the previous study were fed a concentrate diet, their rumen pH was higher than the average for steers utilized in our study (6.61), ranging from 6.83 to 7.03 [31]. As the antimicrobial activity of thymol increases under pH conditions closer to 5.5, based on in vitro estimates [51], dietary conditions in the study with rams [31] did not create a rumen environment that would alter the antimicrobial efficacy of thymol, and diet may have been responsible for the observed treatment differences. This indicates that the effect of thymol depends on basal diet composition and ruminal pH, explaining inter-study variability.

We observed a quadratic increase in A:P, with the peak observed for steers receiving 240 mg thymol/kg intake. We also observed a linear increase in acetate proportions in accordance with increasing thymol dose. Although modest in magnitude, these increased acetate proportions parallel a numerical, linear increase in forage intake associated with increasing doses of thymol and likely reflect subtle shifts in fibrolytic microbial activity associated with the fermentative substrate rather than the broad antimicrobial activity of thymol. Similar responses in acetate have been documented in vitro and in vivo, where low or moderate EO or thymol doses modulated microbial activity without affecting total VFA concentrations [24,29]. These studies suggest that dose-dependent, selective effects on certain microbial groups, including the partial inhibition of propionate-producing microbes or shifts in H-utilizing pathways, may increase acetate proportions under forage-based and neutral rumen pH conditions. In contrast, previous work did not document the effects of thyme EO or thymol on molar proportions of acetate [32,35], and in vitro research documented decreased A:P when thymol was provided between 50 and 500 mg/L rumen fluid versus no thymol [24,44]. Discrepancies between our results and these studies are likely explained by differences in the fermentative substrate. Diets in Ref. [35] contained a high proportion of grain (>50%), whereas our study utilized a forage diet, indicating that thymol may perform differently depending on the basal diet. Further, Ref. [32] also provided less thymol (50 mg/kg intake) than the current study (120–480 mg/kg intake), which may explain why they did not observe a measurable effect of thymol on molar proportions of VFA. The antimicrobial potency of thymol is pH-dependent, with stronger inhibitory activity reported at lower pH values generally associated with concentrate feeding [26]. Therefore, the relatively neutral rumen pH for our steers may have attenuated the suppressive effects of thymol on microbial populations that produce propionate. Cumulatively, the VFA profile and patterns observed in our study are consistent with both the rumen pH context and fermentative substrate utilized.

In comparing our data with others where beef steers were fed a forage diet [41], we observed similar levels of ammonia-N and a similar rumen pH, confirming that our data are in line with expected values for cattle consuming forage. However, total VFA in the current study (80.2 to 84.9 mM) was lower than that of previous work (94.3 to 97.8 mM) [41]. This is likely due to the low CP content of the forage in the current study (3.4% as compared to 6.6% in other research [41]) and concomitant decreased forage degradability, rather than a reflection of thymol. This interpretation of our findings aligns with well-established relationships between ruminally available N, microbial growth, and the extent of fermentation.

Ionophores (e.g., monensin), when fed to cattle consuming forage, and red seaweed added to a forage substrate in vitro, decrease ruminal A:P, thus increasing rumen efficiency and decreasing enteric methane [52,53]. Propionate is energetically favorable from a production standpoint, thus making these feed additives arguably more advantageous than thymol, which quadratically increased A:P in our study. However, propionate proportions were not significantly affected by thymol, and A:P began to decline for steers consuming the highest dose of thymol, indicating that higher doses may exhibit different effects on molar proportions of VFA from those that we observed at the doses utilized in this study. If the mode of action of thymol is diet-dependent, as suggested by previous research, its effects under high-concentrate feeding may differ substantially from those observed under forage-fed conditions. In vitro data [35,51] indicate that thymol may increase ruminal propionate proportions when more concentrate is present in the diet, thus potentially improving the energetic efficiency of rumen fermentation and performing similarly to ionophores and red seaweed. Future studies should evaluate thymol in concert with concentrate-based rations to determine if these propionate-enhancing effects can be replicated in vivo.

Determining a thymol dosage that does not inhibit intake or rumen fermentation is important due to in vitro research demonstrating the antimicrobial potency of this compound [26]. As the thymol doses provided in this study did not affect cattle acceptance (up to 330 mg/kg intake) or diet utilization (up to 480 mg/kg intake), it would be beneficial to assess thymol at higher doses to determine a maximum threshold beyond which diet utilization diminishes. Because we did not observe negative effects at the doses provided in this study, the maximum tolerable dose remains unidentified and should be a focus of future work. Identifying the threshold beyond which thymol impairs intake, digestion, and ruminal fermentation is critical for future adoption as a feed additive in forage- and concentrate-based beef feeding systems.

## 5. Conclusions

These study findings provide novel and relevant knowledge of the effects of increasing levels of dietary thymol in beef steers consuming forage. Ultimately, thymol can be provided to cattle consuming forage up to 330 mg/kg intake and 480 mg/kg intake without negatively affecting acceptance or diet utilization, respectively. Study limitations are analytical challenges that reduced the sample size of adipose tissue to be analyzed for aromatic volatile characteristics, limiting our ability to interpret those data with confidence. As we utilized 4 × 4 Latin Squares for our research, which are established experimental models, the *n* is relatively low, and replication across a larger sample size is warranted. Further, as forage utilization and fermentation were not depressed in this study, we cannot determine the threshold beyond which dietary thymol inclusion would be detrimental to animal performance.

Future research should investigate the effects of higher doses of thymol on intake and digestion while still considering practical aspects of production, such as economics and acceptance. As there is potential that thymol may differentially affect rumen fermentation if a concentrate ration is used rather than the forage diet used in this study [51], future research should also evaluate the effects of thymol in cattle fed different diets, including total mixed rations.

## Figures and Tables

**Table 1:** Chemical composition of basal diet provided in experiment 1 and experiment 2.

Experiment 1: Chemical composition, %DM ^1^			
Item	Hay	Alfalfa cubes	Cottonseed meal
OM	90.4	89.0	93.1
NDF	75.6	46.2	35.3
ADF	43.3	34.6	24.0
CP	7.20	18.4	43.0
Experiment 2: Chemical composition, % DM			
	Hay	Alfalfa cubes	---
OM	89.6	86.7	---
NDF	69.9	35.4	---
ADF	49.3	27.8	---
CP	3.44	17.9	---
Calcium	0.35	1.71	---
Phosphorus	0.04	0.24	---
Potassium	1.27	2.87	---

^1^ DM: dry matter; OM: organic matter; NDF: neutral detergent fiber; ADF: acid detergent fiber; and CP: crude protein.

**Table 2:** Intake of alfalfa cubes soaked in increasing concentrations of thymol by beef steers (*n* = 4) consuming forage (experiment 1) ^1^.

Treatment	Treatment Intake, kg/d ^2^	Hay Intake, kg/d ^3^
0 mg thymol/kg forage intake	1.14	9.78
110 mg thymol/kg forage intake	1.10	9.99
220 mg thymol/kg forage intake	1.11	10.1
330 mg thymol/kg forage intake	1.06	9.25

^1^ Alfalfa cubes were provided daily at 0.20% BW, and thymol was dosed based on individual animal forage intake from the previous three days. ^2^ SEM ± 0.03; effect of treatment × day: *p* = 0.51, treatment: *p* = 0.17, day: *p* = 0.40, and period: *p* = 0.26. ^3^ SEM ± 1.42; effect of treatment × day: *p* = 0.71, treatment: *p* = 0.18, day: *p* ≤ 0.01, and period: *p* = 0.35.

**Table 3:** Effect of four graded levels of thymol, including a control, on intake and digestion in beef steers (*n* = 4) consuming forage and alfalfa cubes (experiment 2).

	Treatment					Contrast *p*-Values	
	CON ^1^	120-T	240-T	480-T	SEM	Linear	Quadratic
*n*	4	4	4	4			
OM intake, kg/d ^2^							
Forage	4.48	4.58	4.63	4.74	0.31	0.87	0.55
Supplement	1.01	1.02	1.07	1.09	0.07	0.96	0.23
Total	5.49	5.59	5.70	5.82	0.38	0.89	0.47
Digestible	3.15	3.01	3.24	3.32	0.18	0.54	0.31
Total tract digestion, %							
DMD	53.2	49.3	52.3	53.0	2.86	0.22	0.52
OMD	57.8	54.4	56.8	57.8	2.81	0.28	0.57
NDFD	64.0	60.7	62.1	65.3	2.49	0.19	0.51

^1^ CON: no thymol, 120-T: 120 mg thymol/kg intake, 240-T: 240 mg thymol/kg intake, and 480-T: 480 mg thymol/kg intake. ^2^ OM: organic matter; DMD: dry matter digestibility; OMD: organic matter digestibility; and NDFD: neutral detergent fiber digestibility.

**Table 4:** Effect of four graded levels of thymol, including a control, on ruminal fermentation in steers (*n* = 4) fed forage and alfalfa cubes (experiment 2).

	Treatment ^1^					Contrast *p*-Values	
	CON	120-T	240-T	480-T	SEM	Linear	Quadratic
*n*	4	4	4	4			
Ammonia-N, mM	2.42	1.87	2.10	2.28	0.21	0.83	0.09
Total VFA ^2^, mM	81.9	84.9	80.2	84.7	3.55	0.83	0.83
Molar proportions, %							
Acetate	74.8	74.9	75.4	75.0	0.14	0.04	0.08
Propionate	14.5	14.4	14.1	14.4	0.17	0.33	0.09
Butyrate	8.40	8.30	8.30	8.30	0.17	0.46	0.74
Isobutyrate	1.10	1.10	1.10	1.10	0.02	0.90	0.79
Valerate	0.60	0.50	0.50	0.50	0.02	0.12	0.52
Isovalerate	0.70	0.70	0.60	0.70	0.03	0.32	0.85
Acetate: Propionate	5.18	5.20	5.38	5.21	0.07	0.21	0.05
pH	6.59	6.59	6.63	6.61	0.04	0.62	0.83

^1^ CON: no thymol, 120-T: 120 mg thymol/kg intake, 240-T: 240 mg thymol/kg intake, and 480-T: 480 mg thymol/kg intake. ^2^ VFA: volatile fatty acid.

## Data Availability

The original contributions presented in this study are included in the article/Supplementary Material. Further inquiries can be directed to the corresponding author.

## Supplementary Materials

The following supporting information can be downloaded at: https://www.mdpi.com/article/10.3390/ani15243637/s1, Table S1: Subcutaneous adipose tissue aroma volatile concentrations (ng/g muscle) for four graded levels of thymol including in steers (*n* = 4) fed forage and alfalfa cubes as detected by GC/MS analysis (experiment 2).
