# Supplementary material for: Diet Acceptance and Utilization Responses to Increasing Doses of Thymol in Beef Steers Consuming Forage
Source: Animals (Basel). 2025 Dec 17;15(24):3637. doi: 10.3390/ani15243637 (PMC12729586; doi:10.3390/ani15243637)
Supplement: Supplementary file 1 [file animals-15-03637-s001.zip › animals-3983892-supplementary.pdf]

Supplemental Materials

Supplemental Table S1. Subcutaneous adipose tissue aroma volatile concentrations (ng/g muscle) for four graded levels of thymol including in steers (*n*=4) fed forage and alfalfa cubes as detected by GC/MS analysis (experiment 2)[1]

	Treatment[2]				
	CON	120-T	240-T	480-T	SEM
n	4	4	4	3	
2-propanol	2.03	3.45	1.09	0.01[3]	1.82
Acetic acid	0.93	0.41	0.63	2.01	0.12
2-Butenal	0.40	1.91	0.84	0.43	0.68
Benzene	0.19	0.22	0.22	0.29	0.12
1-Penten-3-ol	1.74	0.59	1.62	0.01	1.29
Pentanal	7.99	9.05	7.83	6.04	3.01
(S)-Hept-6-en-ol	0.25	0.01	0.01	0.01	0.10
Butanoic acid, methyl ester	0.25	0.19	0.29	0.17	0.08
2-Butenal, 2-methyl-	0.85	0.76	0.60	0.51	0.41
2-Pentenal, (E)-	1.49	1.36	0.79	1.17	0.77
1-Pentanol	0.67	0.28	0.48	0.32	0.36
Toluene	0.75	0.40	0.39	0.01	0.22
2-Hexanone	0.16	0.08	0.07	0.04	0.09
Hexanal	27.8	24.2	29.4	36.3	5.63
2-Pentanone, 4-hydroxy-4-methyl-	9.69	3.25	7.09	5.25	3.99
2-Hexenal, (E)-	0.76	0.55	0.55	0.30	0.26
2-Heptanone	0.85	0.58	0.67	0.86	0.23
4-Heptenal	0.77	0.12	0.23	0.21	0.29
Heptanal	14.03	7.72	7.19	19.44	4.52
Hexanoic acid, methyl ester	0.75	0.10	0.11	0.33	0.45
4-nitrophthalamide	0.07	0.08	0.29	0.08	0.11
Formamide, N,N-diethyl-	2.93	5.46	6.51	15.64	1.44
2-Heptenal, (E)-	1.69	0.97	1.09	0.68	0.68
2-Heptanone, 6-methyl-	0.45	0.31	0.37	0.62	0.10
Benzaldehyde	1.05	0.58	0.85	1.09	0.24
n-Caproic acid vinyl ester	28.0	14.9	23.6	29.8	7.17
Furan, 2-pentyl-	0.83	0.22	0.47	0.37	0.31
2,4-Heptadienal, (E,E)-	0.84	0.44	0.38	0.73	0.31
Octanal	2.37	1.00	1.15	2.74	0.62
dl-Limonene	0.03	0.02	0.06	0.24	0.03
E-2-octenal	0.37	0.32	0.22	0.22	0.24
3,5-Octadien-2-one, (E,E)-	0.86	0.31	0.45	0.36	0.30
Acetophenone	0.19	0.01	0.01	0.19	0.07
Nonanal	4.64	1.66	2.24	3.77	1.26
2,5-Dimethylcyclohexanol	0.31	0.11	0.36	0.50	0.12
Nonenal	1.33	0.08	0.40	0.42	0.40
Benzenamine, 2,5-dimethyl-	9.72	5.11	7.64	7.29	2.76
2,4-Nonadienal	0.14	0.01	0.01	0.01	0.05

Benzene, 1,3-bis(1,1-dimethylethyl)-	0.61	0.69	0.82	1.10	0.28
2-Decenal, (E)-	0.11	0.01	0.03	0.03	0.51
Nonanoic acid, ethyl ester	0.16	0.01	0.18	0.02	0.14
Xycaine	23.3	86.0	37.8	69.5	23.9
Propanal	0.36	3.11	1.01	2.27	0.87
Acetic acid, methyl ester	0.17	0.23	0.08	0.61	0.89
Butanal, 3-methyl-	0.08	0.01	0.09	0.31	0.05
Propanoic acid	0.38	0.01	0.15	0.74	0.18
Benzene, methyl-	0.12	0.49	0.15	0.62	0.16
2-Furancarboxaldehyde	0.01	0.06	0.01	0.11	0.03
Decanal	0.08	0.09	0.14	0.13	0.05
2-Propanone	2.63	1.19	2.85	1.75	1.46
Disulfide, dimethyl	0.03	0.23	0.29	0.08	0.14
Butanoic acid, 2-methyl-, methyl ester	0.12	0.03	0.01	0.01	0.04
Benzene, 1,2-dimethyl-	0.10	0.09	0.21	0.01	0.05
4-Methylpent-1-en-3-ol	0.33	0.24	1.65	0.67	0.73
Decane	0.45	0.12	0.18	0.13	0.13
Hexenal, 2-ethyl-	0.10	0.02	0.21	0.09	0.07
Ethanone, 1-phenyl-	0.01	0.08	0.06	0.01	0.05
Dodecane	0.30	0.06	0.12	0.01	0.18
Butanal	0.01	0.25	0.28	0.09	0.12
Styrene	0.46	0.65	0.23	0.33	0.32
2-Octanol	0.70	0.11	0.35	0.28	0.36
Nonanoic acid	0.02	0.03	0.01	0.02	0.01

[1]The lowest detectable level of aroma volatile was 0.01, and only aroma volatiles which had detectable levels present in >2 treatments and >3 samples within treatments were included. Thymol was only detected in two samples and was excluded.

[2]CON: no supplement, 120-T: 120 mg thymol/kg intake, 240-T: 240 mg thymol/kg intake, 480-T: 480 mg thymol/kg intake
